# "The Hospital" Nursing Mirror

**Published:** 1900-12-29

**Authors:** 


"The Hospital" Nursing Mirror.

Being the Nursing Section of "The Hospital."

[Contributions for this Section of "The Hospital" should be addressed to the Editor, "The Hospital" Nursing Mirror, 28 & 29 Southampton Street, Strand, London, W.C.]

### Notes on News from the Nursing World.

### OVERWORKED ARMY SISTERS IN SOUTH AFRICA.

It would seem that the arrangements in regard to the distribution of the nursing staff available in South Africa are still extremely defective. About this time last year six Princess of Wales's nurses went out to the seat of the war, and we hear that five of them are at the present time *hors de combat*. Sister Davidson left No. 6 General Hospital, Johannesburg, for England about the 20th of this month. Sister Asman has been invalided home, and is expected to arrive shortly. Sister Skillman, of St. Bartholomew's, who, as we mentioned last week, nursed Miss Roberts through her illness, is returning with Lord Roberts and his family. Sister Peers and Sister Lightfoot have both been very ill, but are now convalescent. The sixth sister is also, we understand, perilously near illness. This is a state of things which surely might have been prevented with only the exercise of the most ordinary precautions. The six sisters have been constantly at work since they landed in South Africa last February, and all nursing sisters find the air of Johannesburg very enervating. It would occur to most people that in such circumstances the sisters who have been sent out for a few months only, and have had little or no work, should be assigned duty at places like Johannesburg to relieve those who have been engaged practically all through the war. This course would appear to be dictated by motives of humanity as well as of economy. It stands to reason that the opposite policy, involving a continuous strain, knocks up the majority of women, and, if persisted in, must ultimately have the effect of placing all on the sick list. At any rate, the fact that five out of six of the Princess of Wales's nurses have been incapacitated in less than twelve months points to a singular want of administrative ability on the part of the authorities who direct the nursing staff and arrange their duties and reliefs. In the interests alike of sick and wounded soldiers and of nurses, we call the attention of the War Office to the urgent need which evidently exists for a better control of the Army Nursing Service at the centre of operations in South Africa.

### FRENCH NURSES IN SOUTH AFRICA.

It has been suggested that the Queen should recognise the services of Madame de Ferrières and her staff of nurses in Johannesburg by bestowing upon the former some mark of her favour. Madame de Ferrières is the head of the French Hospital established in Johannesburg at the end of last May, and, whatever the original intention at that period, British and Boer soldiers alike have since been tended with devotion by the nurses, who were drawn from the staff of the Convent of the Sacred Heart. We do not doubt that care will be taken in the proper quarters to bring before Her Majesty the nature of the work done in the hospital. English nurses would be the last to grudge French members of the same profession any honour which Her Majesty may please to confer upon them.

### THE VICTORIA NURSES' INSTITUTE AT CAPE TOWN.

The annual report of the committee of the Victoria Nurses' Institute at Cape Town is particularly interesting just now. The matron, Miss M. L. Damon, states that 23 nurses have joined the Institute since October 1899, two have resigned to be married, and one died of enteric at De Aar while nursing for the railway, 18 went home on transports with the wounded, two of these going twice. There are 13 still nursing at the front: one was in the Bourke's Red Cross Hospital at Pretoria from the beginning of the war; and another was nursing in Ladysmith throughout the siege. The total number on the books is 78, but many of these are scattered about in different parts of the country; some have married, some have died, some have returned to England; so that the actual working staff, including those now with the troops, is from 38 to 40. "The Army Nursing Reserve have now," adds the matron, "signified their willingness to receive some of our nurses; two have already got their A.N.R. certificates and medals, and five more have sent in applications." We are glad to see that the Institute is now self-supporting, though the committee have still to appeal for public help from time to time in order to obtain funds for necessary repairs to the building in Hof Street.

### CHRISTMAS AT THE LONDON HOSPITAL.

The Christmas festivities at the London Hospital took place this year before Christmas. It was thought by the governing body that the previous Saturday would be more generally convenient, and accordingly the afternoon and evening of that day were given up to the entertainment of the patients, the wards being visited, between four o'clock and closing time, by bands of students, known respectively as Crocks, Musketeers, Magpies, and Stop Gaps. Dressed appropriately so as to represent their names, they gave songs, humorous recitations, dances, and short sketches, all of which created great diversion and elicited enthusiastic applause. The nurses and the medical and surgical staff, of course, rendered untiring assistance in providing for the comfort and enjoyment of the patients. An interesting feature of the day was the placing of a memorial clock in the Mellish Ward to the memory of a former sister, who left the London Hospital to serve in South Africa, and died at the front from typhoid fever. This memorial was much admired.

### CHRISTMAS AT ST. GEORGE'S HOSPITAL.

The large pile of evergreens in the vestibule of St George's Hospital on Christmas Eve gave evidence of decorative intentions; and our representative was informed that holly and other evergreens are used in the wards, but no coloured paper. Enough toys have been received from *Truth* to enable each child in the hospital to have a present and a lady has sent a kind and thoughtful gift in the shape of bottles of Eau de Cologne, of which there are sufficient for each patient. The patients' Christmas tea is on Friday, and there are Christmas trees in preparation for the children. On January 5th an entertainment will be given in the Board-room, when all who are well enough to come down will do so; a large number will be carried from the wards. The entertainments for the nursing staff will take place on January 17th and 18th.

### THE JOWITT PENSION FUND: A SUGGESTION.

A movement has been initiated in Leeds, having in view the recognition of the services of Mr. Benson Jowitt, the treasurer of Leeds Infirmary for nearly twenty years, and an unfailing friend of nurses. At an influential meeting the other day it was decided to establish a Jowitt Pension Fund for the benefit of the nurses of the Infirmary, and a committee was formed to carry the decision into effect. The chairman calculates that a sum of between £2,000 and £3,000 will suffice, but, perhaps, before the Leeds Infirmary governors decide upon the details of the scheme they might find it worth while to communicate with the Society of the Royal National Pension Fund for Nurses. The affiliation of the Jowitt Pension Fund with the Royal National would give the nurses it is sought to provide for the maximum of advantages for the minimum of cost, and would materially lesson the work of the managers of the former.

### RULES IN PRIVATE NURSING.

The question raised by our correspondent "Watcher" continues to excite considerable interest. We have always advised nurses who desire to engage in private nursing to ascertain, before they accept an appointment on the staff of an institution, whether there is a committee. In that case a list of rules can probably be obtained, and the nurse can find out for herself what duties she is expected to undertake. Prevention is better than cure, and in a well-ordered private nursing institution controlled by a committee there is no risk of nurses being asked to render services which cannot be performed without the sacrifice of modesty.

### ANOTHER COMPLIMENT TO THE WINDSOR INFIRMARY STAFF.

The general manager of the Great Western Railway has sent a cheque for 200 guineas to the Windsor Infirmary. It was voted as a special donation to the funds in consideration of the expenses thrown upon that institution through the unfortunate accident at Slough in June. Mr. Wilkinson, in his letter, expressed the company's appreciation of "the valuable services rendered by the authorities of the infirmary, and of the kind sympathy and attention which the sufferers from the accident received at the hands of the infirmary staff." We congratulate the matron and nurses upon the manner in which their ministrations have been recognised.

### NURSING IN BEDFORDSHIRE.

There is no lack of influential support to the nursing movement in Bedfordshire. At the annual meeting of the Rural Nursing Association, Lady St. John occupied the chair, and the speakers included the Duke of Bedford, Lord St. John, Canon Haddock, Mr. Whitbread, and the Rev. Paul Wyatt. Yet Mr. Wyatt had to express his disappointment at the small number of subscribers. It is the old story of the few doing their duty and the many neglecting it. The report of the association, and all the speeches at the meeting, showed that the work of the nurses is most satisfactory and that the villagers greatly appreciate it. But, as Mr. Whitbread said, it was hardly anticipated that the central organisation would experience a difficulty in obtaining funds, and the paragraph in the report to the effect that "the difficulty is becoming rapidly more acute, for not only will it become necessary that nurses should be trained with a view to supplying the needs of newly formed districts, but nurses will also have to be provided to fill the places of those whose agreements have expired," should have the effect of rousing the public in the county to a sense of their duty in the matter.

### MORE TROUBLE AT BRIDGEND WORKHOUSE INFIRMARY.

The master of the Bridgend Workhouse having been accused of causing the frequent resignation of nurses, which is still going on, has retorted that the fault is that of the Guardians, who ought, he asserts, to get the nurses to see the "house" before they consent to accept an engagement. According to the master, the salaries offered are liberal enough to tempt hospital nurses to apply for vacancies, but when they arrive and find out the duties they have to perform, and the accommodation offered, they resign. The master further makes the remarkable statement that "the duties are not those which professional nurses are expected to do, but for women who are not particular as to what work they are asked to perform." It is a curious commentary on the master's statements that at a subsequent meeting of the Guardians it was reported that two nurses who had applied for an appointment in the "house" and had been requested to personally appear before the Board, did not do so.

### THE STORY OF A DUM-DUM.

A nurse sends us an interesting story of a bullet. A few days ago she received by post, from the Base Hospital at Naauwpoort, an ordinary match-box containing a dum-dum bullet which had been worked out to form a pencil-case. Enclosed was a note written from an ex-patient in pencil, in very shaky handwriting. It appears that about four years ago whilst working on a ship as an A.B. he was sent aloft to paint the mast. Knowing the mainstay of one of the masts was insecure, he remonstrated with the captain, but was ordered to complete his work and obeyed, with the result that the support gave way and he fell heavily to the deck, sustaining a fractured skull. At the hospital an operation was found necessary, and was performed successfully, but a few months later he became subject to epileptic fits, and after further treatment he was discharged as incurable. Being unable to work, he was prevailed upon by the Sailors' Union to sue the owners of the ship for damages. At the same time he vowed that if he lost the case he would end his life, which was, he declared, worthless in his present condition. About sixteen months ago the case was heard, and the verdict given for the owners, whereupon a sensation was caused by the plaintiff carrying out his resolve, and shooting himself in court. He was at once taken back to his former hospital, where all efforts to locate the bullet were for a long time futile, till our correspondent happened to see the bullet, which had worked its way up under the skin. It was immediately removed, and in gratitude the patient wished to give the bullet to his nurse for a souvenir. The surgeon, however, who had taken great interest in the case, expressed a desire to have it, and it became his property. The sailor completely recovered, and was discharged. He entered the army and was sent to South Africa. But before sailing he said laughingly to the nurse that he hoped the Boers would be better shots than he was, but if not, he would send her the bullet, should he only be wounded, in place of the one which had been removed from him in the home hospital. That promise he has now ratified.

### AN INCIDENT IN THE PRINCIPALITY.

A NURSE who is attending a private case in an inaccessible part of Wales recently had an amusing experience. Her patient being seriously ill, she managed to procure a water-bed, and having half filled it with water she asked a young and very Welsh servant to bring her a pair of bellows. The girl looked greatly puzzled; so the nurse explained what she had done already, and how she wished to inflate the other half of the bed with air. Still silence on the part of the maiden accompanied by a totally incomprehensive stare. The nurse then proceeded to a further and more detailed explanation, describing the appearance of a pair of bellows, its ordinary use, and so on. "I'm sure you must have one in the kitchen," she concluded. At last the Welsh girl was beginning to understand! Deep in thought she stroked her chin slowly and deliberately; then a look of intelligence dawned upon her face, and she spoke:—"Inteed an' for sure, Miss, there iss nothing off the ssort in the housse at all, put how'd 't pe, look you now, to fill the other haf the ped wi' paper?"

### A NEW NURSING HOME AT NAPLES.

SOME interesting particulars reach us of a new nursing home at Naples, which is to be worked as much as possible on English lines. It is called the Villa Regina Natalia, and the lady superintendent is Miss M. Amy Turton. Her fixed staff at present consists of nine hospital-trained Italian nurses—women who for the last five years have been trained in the big Roman, Neapolitan, or Florentine hospitals, under the superintendence of Miss Baxter, a graduate of the J. Hopkins Hospital, Baltimore, and Miss Turton herself, who received her training at the Royal Infirmary, Edinburgh. English-speaking patients are free to call in any of the English or American nurses living in Florence, and they can be attended by their own physician. We observe from the regulations forwarded to us that no patient is accepted without the authority of one of the six members of the medical direction.

### PARISH COUNCILS AND THE SUPPLY OF NURSES.

THE Executive of the Sutherland Benefit Nursing Association, having recently received an application from a Parish Council as to the terms on which they could supply nursing for paupers during illness, have replied that a nurse will be supplied on the request of the inspector of poor and the parish medical officer if the Parish Council pays in advance an annual subscription of 1*s*. 6*d*. for each pauper on the roll at the date of admission to the association, and on August 1st annually thereafter, not including paupers in the combination poorhouse, nor those resident outside the county. Fees will be charged at the rate of 2*s*. 6*d*. for every week that any one pauper may require the services of the nurse. In all cases Parish Councils must supply board and lodgings for the nurse when attending pauper patients, to the satisfaction of the local committee of the association. The committee also pointed out that under the existing Poor Law Acts the Parish Councils have power to provide nursing for paupers during illness. We do not doubt that the example of the Parish Council of Dornoch, who have subscribed for all the poor resident in parish on the terms of scheme of the Sutherland Benefit Nursing Association, will be followed elsewhere.

### COLLAPSE OF A NURSING ASSOCIATION.

THE Thrapston, Islip, and District Nursing Association has come to an untimely end owing, as it appears from the report of the annual meeting, to lack of funds and to its failure to command the sympathy of Thrapston medical men. According to the report of the general committee, the Thrapston doctors have almost ignored the nurses of the association and, being asked to see a deputation on the subject, declined. We share the surprise expressed by Mr. Kidner at this state of things. Mr. Kidner says that "at Rushden, Raunds, and many other places the nurses and doctors appear to work together in a most amicable way, to the manifest advantage of the community at large," and the experience of the Northampton towns and villages to which he refers is certainly the rule in all parts of the country. We can only conclude that either the nurses of the Thrapston Association, or the Thrapston doctors, are unreasonable. At any rate, it is nothing short of a scandal that failure of co-operation between the members of two professions, whose interests and aims are identical, should help to bring about the result of even a temporary suspension of admittedly valuable work. Better counsels will, we hope, prevail, and a society, whose operations have included a town of the size of Thrapston and eight important villages, be revived and put upon a sound basis at an early date.

### NURSING IN MID-CORNWALL.

THANKS to the energy of the Cornwall County Nursing Association, the parishes of St. Stephen's-in-Branwell and St. Dennis are now provided with a trained nurse, who has just taken up her residence at Nanpean, the most central position in the district. The cost of maintenance for a year is £60, and there does not seem to have been much difficulty in raising the necessary amount. There is no doubt that the addresses which have been given from time to time in the county on the benefits of resident trained nurses have stimulated interest and support.

### SHORT ITEMS.

MRS. GEORGE CORNWALLIS-WEST has received a cable from Hong Kong to the effect that the *Maine* left that port on December 1st, bound for England, *via* Suez, Port Said, and Malta, arriving at Southampton about January 10th. After consultation with the naval and military authorities in China, the Government decided to order the ship home, the need for her being no longer felt.—Two vessels have arrived at Southampton with sick and wounded during the week—the hospital ship *Simla* and the s.s. *Orotava*. The former had on board Superintending Sister Drury, A.N.S., Sisters Kirkbride, Bindloss, and Goold, A.N.R., and Sisters Webster, Hiatt, and Littlecott, C.N.S. On the *Orotava* were Nursing Sisters Harland, Cobbold, McLeish, Young, Suttaby, and Rennie.

### Lectures on Medicine to Nurses.

By H. A. LATIMER, M.D. (Dunelm), M.R.C.S. Eng., L.S.A.Lond.; Consulting Surgeon, Swansea Hospital; Past President of the South Wales and Monmouthshire Branch of Brit. Med. Assoc.; Past President of the Swansea Medical Society; Hon. Life Member of the St. John Ambulance Association, &c.

### TREATMENT OF FEBRICULA. MEASLES.

FROM what I have been saying to you about the causes and nature of simple continued fever, you will see that the diagnosis and treatment of the same consists in the recognition of some disturbing cause of a temporary character, which is to be dealt with by simple and appropriate measures. Should the attack be due to disturbance set up by worms in the stomach or intestines, one or other of the drugs which kill or expel these parasites will be administered; should it arise from teething, or constipation, or indigestion, or the catching of a cold, the measures which are practised for the cure of those ailments will be adopted. One thing is certain: we have advanced so far on the road of progress that the invalid who now comes under medical treatment for slight ailments is not likely to be injured by doing so, as was very likely to have been the case in the old days, when starving and bleeding patients were in vogue. For at present a patient suffering from feverish symptoms will be sent to bed if under medical advice, and will be advised to take a light but nourishing diet of farinaceous foods and of broths; he will be allowed to assuage his thirst by drinking freely of some mild diluant, such as barley water, toast water, milk and soda, or plain water, and medicines of a suitable kind for the relief of the feverishness and of its cause will be administered. If in doubt as to the real nature of the attack of illness from which he is suffering, little more than this will be done, and the medical attendant will not hesitate to tell the friends that he cannot definitely pronounce what is the matter with his patient, and that he will have to wait awhile for more definite symptoms to declare themselves before he can give an absolute opinion as to what is the real cause of the illness he has been called in to treat. He will not do as the American physician is said to have done in a case in which he was in doubt. Here, being called in to diagnose and treat an illness in a child which he could not discover the nature of, he is reported to have said, "Waal, mam, I can't say what the little beggar is suffering from, but you give him this powder, which will bring on fits—and I'm a whale at fits." Thanks be! we have now arrived at times when to call for a suspense of judgment in these affairs is not deemed to be making a confession of ignorance, and we are no longer called upon to adopt heroic measures for the quelling of fevers. We are still expected to administer more medicines than are necessary, for people seem to think that nothing is being done for them when ill, unless they are being dosed with pills, powders, or mixtures; but patients have fallen on pleasant days, in comparison with the past, with respect to drugs and other treatment which were formerly nauseous, strong, and often deleterious in their effects. I sometimes think that foreigners—and especially the French—have a better method of treating these light fevers than we have. They are skilled in the preparation of herbal teas, which they serve up hot under the name of "tisane"; and little more than this mild febrifuge, with confinement to bed, is done for such cases as the ones I am speaking of. As a matter of fact, for feverish attacks of a simple nature, very little more than rest, a lighter diet than usual, and a mild aperient are called for as methods of treatment. In itself, rest in bed is curative to the feverish state; by it the excited action of the heart is abated, equal distribution of blood over the surface of the body is promoted, wear and tear of tissue is lessened, and an insensible perspiration takes place, by which heat is reduced. So this is one of the first measures I advocate in febrile attacks of all sorts. I am sorry to say it is by no means a favourite one for adoption by people at large; the majority of persons, according to my experience, hate the idea of being sent to bed, and fight against it with every argument they can call up to their aid. One favourite assertion is that "the bed weakens them." They seem to confound the debility resulting from illness with the remedy adopted for its cure. Lying in bed undoubtedly does cause a feeling of relaxation when indulged in for a while, but it does not really weaken a person. The sense of fatigue induced by it is due to want of use of the muscles, and wears off in a few hours when a little exercise is taken again. You must often have heard people say, in reply to questions by their medical attendants as to whether they feel better and have improved appetites when in bed and recovering from illness, "How can I get stronger and improve in appetite when kept in bed?" and must have noticed how very common it is for appetite and strength to improve when people are under these conditions. The truth is that, in the vigour of life, on removal of disease all the phenomena of health quickly assert themselves, and it is quite a common thing to find strength rapidly regained and the appetite become quite keen with people who are still kept, for precaution sake, in bed. Many of the cases of febricula you will meet with will occur in children. Here you will take the temperatures by placing the thermometer in the groin. With the patient lying on the back, move one leg outwards, and carefully place the bulb and adjoining part of the instrument snugly in the groin, and then replace the limb, laying the bended knee over its fellow. Maintain it in this position for the necessary length of time for a proper observation to be made, and on removal you will find a satisfactory record.

Now I will pass on to a consideration of the first of the specific fevers which I have selected as a topic for lecturing on. I take the subject of measles for my purpose. This fever, although it mainly affects children, is one from which people of all ages may suffer, and I presume that it rarely attacks adults because they have been protected from it by having suffered from the disease in early life. That it is by no means a simple and harmless complaint is shown by the mortality tables and by the heavy death rate which it causes in a new and unprotected community. Thus there was a very severe epidemic of it in 1845 in the Faröe Islands: and in 1874, soon after Great Britain took over the Fiji Islands, it was introduced amongst the unprotected natives and quickly assumed quite the character of a plague, 50,000 natives—probably a gross exaggeration—being said to have died of it. I have taken the trouble to look up old copies of the *Lancet* on this question, and I find in quoting from a leading article of that paper—dated June 19, 1875—it stated "during the last two years a remarkable epidemic of measles has passed through the southern half of the globe: and in several countries which had before been entirely, or for a long time, free from the disease, its effects have been very severe, and its tendency to attack unprotected adults almost as readily as children has been very marked. In the latter part of 1873 the disease was introduced into the Mauritius, probably from South Africa, where, as an isolated epidemic, it existed during the previous year. In Mauritius it had been long unknown, and it rapidly spread to every part of the island. During the last quarter of 1873 and the first quarter of 1874 it caused more than 2,000 deaths. . . . In Port Louis in three months 9 per 1,000 died of the disease. . . . Persons of all ages suffered, even up to advanced periods of life, and the number of adults attacked was very considerable . . . 33 per 1,000 of the deaths are given as of persons over five years, and 10 per cent. of the deaths were of adults. In the summer of last year the disease gained a footing in South Australia, where a general epidemic had been unknown for nearly a generation. It spread rapidly among the population both of Adelaide and the surrounding districts. Adults suffered in large numbers." So, you see, in unprotected communities grown-up people are attacked freely by measles; and facts would seem to bear out the correctness of the assertion that the general immunity of adults here is due to their being protected by an attack of the disease during childhood.

### A Christmas Talk with a Patient.

BY A NURSE.

"AIN'T it a nice change like, to be spendin' Christmas in a 'orsepital, instead o' at 'ome? Well, that depends on 'ow yer looks at it. Now, if I was a lonely sort o' a chap without no fam'ly nor friends nor nothin', well I might say as I'd think meself best off bein' in 'ere. But yer see it ain't that way with me, me bein' a fam'ly man, 'avin' a wife and six little ones, and a 'ome of me own. I seems fer to miss 'em like, and to wish as they could 'ave some of the good things what's comin' my way. Makes yer feel sort o' sore in yer inside, in a manner o' speakin', to 'ave good food and kind o' dainty things yerself, and know as them at 'ome is 'avin' worse victuals than usual, through the one as earns the money bein' in the 'orsepital.

"There! it makes me fair sick to think of it sometimes when me tasty bit o' custard pudding comes along, or me soup, or a great mug o' milk, I carn't 'elp rememberin' as Kitty—that's our littlest gal—oughter to 'ave pints o' milk—by what the doctor said—she bein' most white and queer. As fer the bit o' chicken what I've 'ad to-day, bein' Christmas Day, and me a little better—why I tell yer plain, I'd a longin' inside o' me, fit to make me bust, fer to tie up that there chicken, and send it 'ome to 'Lizer, that's me wife. Well, she ain't never tasted nothin' like it in all 'er born days—I'll take me oath o' that.

"Pore old gal, she've a' bin a good wife to me, and she've a' 'ad a rough time since I've laid 'ere, though she don't complain. But it makes a 'eavy pull on a woman when 'er man's away, and she've got to find the rent and everythin' 'erself. Makes yer feel a bit mad to be lyin' 'ere, and thinkin' about yer little 'ome, what yer wife's strugglin' 'ard fer to keep together. She've a' put away most of our bits of things, to git the rent. Well, I ain't sayin' they're valuable things, not such as you might 'ave in your droring-room, but us pore people sort o' 'olds by our sticks o' furniture, same as you might to yours. Give me quite a queer sort o' sensation when Lizer told me as she'd 'ad to let our armchair go. There! I fair loved that armchair. Me and 'er we bought it off of a man down the Old Kent Road, when we was a' courtin' and gettin' our things together. But it ain't no use fer to lie 'ere and grizzle. When 'Lizer comes I makes a effort to grin and seem as if I liked bein' 'ere—makes it less 'ard fer 'er, yer see.

"Oh! it's very cheerful 'ere. I ain't denyin' that; I wouldn't never say a word agin 'orsepitals. God bless them as started them, and them as looks arter them, I say. But yer feels a bit lonesome like now and agin, when you're a fam'ly man. Now you wouldn't never believe 'ow I misses them children. I *am* set on them kids o' mine. And the missus, well, I misses 'er terrible. 'Er and me we've bin married best part o' fifteen years, and when you've bin married all that while, yer feels kind o' lonesome without yer old woman. But it's thinkin' o' 'er strugglin' what 'its me 'ardest. There's 7s. 6d. fer rent, and coals and bread is terrible dear. Makes me 'eart ache for to look at the beautiful blazin' fire in this 'ere ward, and think o' 'Lizer and the kids with a bit of a black fire in the grate, or no fire at all.

"Ain't I felt ill in meself, did yer say? Well, it ain't very nice for me to get smashed up like I was, through a van runnin' over me. Me arm was broke, and me ribs and me lungs got queer on account o' me ribs runnin' into 'em. Narsty sort o' a sickenin' pain I 'ad, shootin' through me like knives bein' put in. Made yer feel as if yer must 'oller out now and agin. But it ain't no use fer to 'oller out—don't make the pain no better, and upsets the rest of the ward like. Wonderful, ain't it, 'ow yer learns to keep still and take things quiet, when yer lays in a 'orsepital ward. Well, it's like this: there's others to think of besides yerself, and some of 'em's a lot wuss nor what you are, and you gets interested in 'em, and forgets yer own pain. There's many a chap in this 'ere ward what's wuss off nor me. There's a old daddy over there what broke 'is leg; and 'e's that blind 'e carn't read, and 'e's orful deaf, and 'e ain't got no friends fer to come and see 'im. Pore old chap! I do feel sorry for that old boy. Now look at me. There's 'Lizer comes to see me, and the kids, and some o' me pals 'll drop in on visitin' days. And now as I'm a bit better I reads a bit—nice picture papers we've got 'ere, and books as would surprise yer—they're that interestin'.

"Oh! it's lonesome sometimes, and I've felt orful queer and bad; but, bless yer, there's good things in 'orsepital, what you mightn't think about till you'd laid 'ere. Fust there's the kindness what yer gets from the doctors and nusses and dressers and all. Well, that's a treat, that is—makes yer 'eart swell with gratitude; makes yer think as things ain't all bad in the world. Yer *do* get despondin' when ye're ill, an' yer wife strugglin' and the winter 'ard like this one is. Lyin' 'ere and seein' everybody so kind cheers yer up, and makes yer think as God ain't forgot yer arter all, and p'raps times 'll git better by and bye.

"There's another thing I'll tell yer what's good in 'orsepital. There's a lot of werry rough coves what comes in 'ere as patients—werry rough coves indeed, but you wouldn't believe 'ow rough they was, seein' 'em lie 'ere so meek, doin what they're told like lambs. Seems as if the discerpline like kind o' makes 'em quiet; never swears, nor nothin' o' that, they don't, which is werry surprisin', when you remembers they ain't no class, and don't think nothin' o' bad langwidge in a general way. No class at all they ain't, but it does 'em a sight o' good fer to 'ave a lot o' lidies lookin' arter 'em, lidies what's kind and gentle, such as they ain't never seen before. Oh! our nusses is kind and good, and they 'as a lot o' power in their 'ands. They can make us rough chaps quiet and well be'aved; they sort o' make yer feel as it's worth yer while to make a kind o' effort arter 'igher things. Nusses oughter always remember what a lot o' power they 'as over us rough folk; it ain't easy fer a stoopid chap like me fer to put it inter words, but there, the nusses often and often sends us out o' 'orsepital better chaps than what we come in.

"Gives yer time to think, bein' ill in a 'orsepital does, makes yer want to turn over a new leaf and start agin, if you've made mistakes. Oh! it does us a power o' good, there ain't no mistake about that.

"Got to go 'ave yer? Well, thank yer kindly fer comin' to talk to me. Oh! yes, I'm always one as 'ull say a 'orsepital's a grand place—a grand place—it makes a better man of yer, as well as curin' yer diseases, that's what I say."

### To Nurses.

WE invite contributions from any of our readers, and shall be glad to pay for "Notes on News from the Nursing World," or for articles describing nursing experiences, or dealing with any nursing question from an original point of view. The minimum payment for contributions is 5s., but we welcome interesting contributions of a column, or a page, in length. It may be added that notices of entertainments, presentations, and deaths are not paid for, but, of course, we are always glad to receive them. All rejected manuscripts are returned in due course, and all payments for manuscripts used are made as early as possible at the beginning of each quarter.

### An Amateur Nurse at the Front.

INTERESTING EXPERIENCES.

MRS. BAGOT is not one of the ladies of whom Mr. Treves complained. She did not go to South Africa with her husband, Captain Bagot, M.P. for South Westmoreland, with the idea of taking upon herself work which she had not qualified herself to perform, or of hampering the authorities by her presence where she was not wanted. Her position is explained in the preface to her book *Shadows of the War*, which has just been published by Mr. Edward Arnold. "When I went to South Africa," she says, "I had not expected or intended to do any nursing, having seen just enough of hospital work to realise my own deficiencies. In going out to a war, however, I have fulfilled a dream of twenty years' standing, and in preparing for it I have seized every available opportunity of acquiring practical nursing experience and getting surgical and medical training." Mrs. Bagot's one object was, in fact, to remain with the Portland Hospital, which she originated, and for which she raised the money, the name being given to it, at her suggestion, because the Duke of Portland contributed the handsome sum of £5,000 to the fund. In the early chapters of the highly-written and extremely interesting account of her experiences, she describes the intense pleasure and relief she felt when the Portland Hospital was established at Rondebosch. As to her own quarters, she says: "For my part, as I gazed with pride at my dear little bell tent, which stood all in readiness, I felt thankful to be alive." In this admirable spirit of high enthusiasm she entered upon her work, enjoying the advantage of having her husband, who was honorary secretary, for a colleague, the nursing sisters of the hospital being Sisters E. Pretty, A. M. Cox Davies, F. Russell, and A. Davies.

NOT A SINGLE STRETCHER.

One of the numerous illustrations by which the book is adorned represents the arrival of waggon-loads of sick and wounded. Naturally, this event created a considerable flutter of interest amongst the staff, but it appears that it also provoked disappointment. "However, it so happened that every single patient was able to walk into the hospital, and the sisters were found almost in tears afterwards, as, they murmured in desponding accents, 'not a single stretcher case has been sent to us.'" And yet there were several bad cases which Mrs. Bagot describes all the more effectively because she uses simple language, and has the power of pathos.

LORD ROBERTS AND HIS MEN.

Mrs. Bagot's account of the visit of Lord Roberts to the hospital affords yet one more proof of the interest he took in all the arrangements for the patients, and of his remarkable tact. "Lord Roberts talking with his men," says Mrs. Bagot, "gave one rather the impression of a friend amongst friends than of a great general going his round merely from a sense of duty. They looked after the erect little general, as with grave considerate face he passed into another tent—fondly of course, and proudly, but many with an expression full of tender sympathy"—the sympathy being due to the fact that, as the author touchingly puts it, he was "fresh from a sorrow which might have undone many younger than him, and straight from the bitterest trial a great man can face."

"LITTLE GOD" KIPLING.

In another chapter the author mentions the impression given by the first appearance at the hospital of Mr. Rudyard Kipling. His presence acted "like steel upon every one of the patients. As he walked into the camp you might have seen men striving and straining to get a peep at him from their beds, whilst other excited faces were looking out from the tent door." And again: "It was an incomparable sight, Kipling sitting like a little god in the midst of the patients, the quaintest figure, dressed in the ugliest of reach-me-down suits, spring-side boots, and a brown slouch hat pulled right over his eyes, with a crumpled-looking face below beaming with fun and good humour." An illustration gives point to this word-picture.

THE QUEEN'S CHOCOLATE.

Of course there is an account of the reception of the Queen's chocolate by the patients in the Portland Hospital. "At sight of the boxes all the men who could do so stood instinctively at attention. It was a gift from their Queen. Those in bed nearly cheered for pleasure and held out their hands delightedly. It may be imagined how much they prized their gift; many of the men dared not so much as untie the little ribbon that bound it, and very few even tasted the chocolate. They usually held the little packet for a while, then, with tender, reverent hands, they wrapped it carefully in a handkerchief and put it in a safe place until they had an opportunity of registering it and sending it to England. 'Everyone at home must have a taste of it,' they said, 'and then we can keep the box as an heirloom.'"

IN A MILITARY HOSPITAL.

At the end of three months Mrs. Bagot quitted her "beloved Rondebosch," and went to Naauwpoort, at that time the most advanced base hospital on the road to Bloemfontein. As she expected to remain there at least a fortnight, she asked herself how to find some work to occupy the time. "These white hospital tents in the distance seemed to give the only reasonable solution to this question," and to the tents she accordingly went.

It is due to both the P. M. O. of the hospital and to Mrs. Bagot herself to mention that she only asked "for some employment of the nurse that lived in the hospital," and we assume that the single reason why she was told she could act as invalid nurse to one of the principal nursing sisters in the surgical division was because, though an amateur, her help at that time was really needed, and therefore welcomed. She, at least, cannot be blamed if the nursing staff then was inadequate, nor yet the sister superintendent, who assented to the engagement.

THE NURSING STAFF.

Mrs. Bagot speaks in warm terms of the manner in which the sisters discharged their duties. Of the superintendent she says that "she carried them out in a way which called forth the devotion of the sisters and commanded the respect of all the staff." She herself was under the orders of Sister B——, of Guy's Hospital, one of the six hospital sisters from London. "In this," she writes, "I soon found out my good fortune. Sister B—— was looked up to as a particularly skilful nurse, but it did not in any way diminish her patience and kindness in making the way clear to me and my duties easier. I tried to atone for my many deficiencies by being implicitly obedient, and I can never be grateful enough for what Sister B—— has taught me." As to her actual work, Mrs. Bagot says: "After the first morning duties in the wards were finished I used to go round with Sister B—— when the surgeon visited the patients, and was presently allowed to assist in the dressing of the wounds. I was also expected to attend the operations performed on Sister B——'s patients." Several of the cases in Sister B——'s wards are mentioned, notably, that of one Irishman who was the victim of a soft-nosed bullet and had to undergo two operations. The operating theatre was under the charge of Sister L——, of King's College Hospital, and Mrs. Bagot says of it "that it was so perfect in its way that it was hard to believe it was nothing but a movable building." An illustration in the volume shows an operation in progress, with sisters in attendance.

A GREAT PRIVILEGE.

How thoroughly Mrs. Bagot appreciated the honour of serving the sisters is shown in the closing passage of this chapter. At length she receives a summons to go back to Bloemfontein, and she obeyed with sorrow. It had been, she thankfully confesses, "a great privilege to her to see the working of a military hospital like this," and she adds: "The memory of that little band of men and women, all working and slaving together so unostentatiously, wholeheartedly, and devotedly, soothing and alleviating sufferings of hearts as well as of bodies, and in many cases laying down their own lives also, will live for ever."

### Preparations of Beef-tea for the Sick.

By HELEN TODD, Matron, The National Sanatorium, Bournemouth.

In cases of severe illness when the patient is unable to take solid food, fluid forms of nourishment must be provided. Generally speaking this will be of three descriptions, milk, eggs, and beef-tea. The first two are, fortunately, natural products, and provided they be fresh and obtained from a trustworthy source, may usually be relied on as being what they are represented to be.

### Chaos in the Mind of the Cook.

With beef-tea, however, the case is widely different, and the article sent from the kitchen may vary from a pale, watery, greasy, and unpalatable liquid to a thick, dark-coloured, rich-looking broth; the difference depending not only on the cook's ability and the quality of the raw material supplied her, but also upon her conception of what beef-tea should be. If we take the trouble to glance through say half-a-dozen of the best known authorities on cooking and nursing we shall not be surprised at the chaos in the mind of the average cook.

### The Chemistry of Beef-Tea.

Before proceeding to compare our widely different authorities on beef-tea, let us consider shortly what may be termed its chemistry. Beef-tea is a fluid preparation which should contain as much as possible of the nutritive elements of the meat from which it is prepared. Now "meat" is the same thing as "muscle," and muscle consists of fibres bound into small bundles by connective tissue, from which comes the gelatine which solidifies the beef-tea when cold. The fibres consist mainly of a proteid substance termed myosin, which forms the nutri-ive part of all meat; this myosin is an albuminoid, soluble to a large extent in dilute saline solutions, but coagulable on heating above 160° Fahr. Other constituents of muscle are water, certain inorganic saline matters, phosphates and potash, and a large number of substances existing in very small quantities which are usually classed together as extractives; these give beef-tea its well-known stimulating properties. There is also in all muscle a variable amount of fat.

### South Kensington and other Teaching.

In this country the South Kensington School of Cookery is generally regarded as the highest authority on culinary matters. Its official instructions for making beef-tea tell us to allow 1lb. of gravy beef to 1 pint of water, cut the beef up small, removing all skin and fat, and put it into a saucepan with the cold water and half a saltspoonful of salt; then stir until it almost boils. (Here let me point out that the boiling point of water is 212° Fahr., and that the albuminoids in meat, which contain almost its whole nourishing properties, coagulate at 160° Fahr.—far below "nearly boiling" point—so that in heating our concoction above 160° Fahr. we effectually prevent the essential elements of nutrition passing from the meat into the beef-tea.) Following the directions, we must now allow the beef and water to simmer gently for an hour; it then has to be strained, allowed to cool, have the fat skimmed off and served—not, let us hope, as a food, for that it certainly is not, the albuminoids being all left behind, coagulated by heat and sealed up in the fibres. According to Sir William Roberts, beef-tea made as above is simply water containing the salines and extractives of the meat, i.e. the stimulating substances, and nothing more; with the exception of a trifling amount of gelatine and fat.

Let us now compare some of the various directions for making beef-tea given during the past fifty years by other recognised authorities on invalid and general cooking, marking how they differ in detail. For this purpose Miss Acton's "Cookery Book" of 1858, Domville's "Nursing Manual," 1881, Mrs. Beaton's issue of 1899, and "The Art of Feeding the Invalid," last edition (undated, but quite recently published) will suffice:—(1) To begin with, the raw material required—Miss Acton prefers rump steak, Domville simply "beef," and the others all agree over "gravy beef," which we may safely take to mean shin of beef; (2) as to the proportion of quantities—Miss Acton and Mrs. Beaton each direct 1 lb. of meat to 2 pints of water, the other three advise 1 lb. of meat to 1 pint of water; (3) the only point upon which all our authorities agree is that the meat must be put into cold water, but it is a little disconcerting to find that (4) the methods of cooking are quite irreconcilable: Miss Acton puts her saucepan by the side of the stove, giving its contents an occasional stir, for two hours; Mrs. Beaton brings her beef and water at once to "boiling-point" (!); Domville allows his to stand for one hour, and the authors of "The Art of Feeding the Invalid" let theirs stand "all night" before being subjected to any heat at all. (5) The length of time required for the actual cooking—Miss Acton "gently boils" her meat and water for 15 minutes, Mrs. Beaton lets hers "simmer" 30 to 45 minutes, Domville places his in the oven for one hour, the South Kensington authorities let theirs simmer for the same length of time, whilst in "The Art of Feeding the Invalid" we are directed to "stand the jar containing the beef-tea in a saucepan of boiling water and let it cook for five hours:" (6) all the cookery books with the exception of the last-named tell us to strain off the liquid at once, the beef-tea being now ready for use.

The authors of that book, however, realising the unsatisfactory condition of their beef-tea as a means of nourishment, advise the addition of some of the coagulated albuminoids by beating up part of the meat after the straining and mixing it again with the liquid before serving.

### The Value of Extracts.

This is practically the course recommended by Sir William Roberts and claimed to be carried out on a large scale in the manufacture of the familiar bovril, with the additional advantage, however, that fresh meat is used instead of that from which the beef-tea or extract has been made. Nutritive extracts of this sort, being necessarily kept up to a fixed standard, are, of course, more reliable than the varying beef-tea of the kitchen. Much labour is also saved by their use, as they merely require the addition of hot water before serving, and hence they are nowadays used to a very great extent in hospitals and private houses. They also effect a very considerable saving in hospital finance, as was proved quite recently at one of the large Metropolitan Poor Law infirmaries when the question of home-made beef tea or bovril was very fully discussed. It was found that, although they happened to have an exceedingly low contract for shin of beef (i.e. 4d. per lb.), by using invalid bovril a considerable saving was effected, whilst, if ordinary bovril were substituted, the saving was of course much greater.

### How the Figures Work Out.

The actual figures were as follows:—To make 28 pints of beef-tea, allowing $\frac{1}{2}$ lb. meat to 1 pint water, 14 lbs. lean meat are required, but the loss on fat, skin, &c., being 20 per cent., $17\frac{1}{2}$ lbs. of beef had to be provided at a cost of 5s. 10d., or $2\frac{1}{2}$d. per pint. If the more usual allowance of 1 lb. meat to 1 pint water be calculated, the cost would be 11s. 8d. per 28 pints, or 5d. per pint. To make the same quantity of bovril 1 lb. $5\frac{3}{4}$ ozs. were required, which, at the contract price of 4s. 3d. per lb. for the unflavoured and less salty "invalid bovril," cost exactly 5s. 9d. per 28 pints, or $2\frac{13}{28}$d. per pint. Ordinary bovril at 3s. 9d. per lb. costs 5s. 2d. for 28 pints, or only $2\frac{3}{14}$d. per pint, whilst it compared advantageously with the usual rather weak infirmary beef-tea. The money saving works out at $\frac{2}{7}$d. and $2\frac{15}{28}$d. per pint respectively.

*(To be continued.)*

### Echoes from the Outside World.

THE pessimists say it is "a black Christmas for us all," but things are not surely so bad as that. Most of us did hope that the war would be over before the joy bells rang in the new century, but the assertion that "it is only beginning" is an obvious exaggeration, dictated by partisanship, or due to ignorance. It is, however, sad that there should be a necessity for sending out more troops, and even a suggestion that a special commissioner should be despatched in order to deal with the situation diplomatically is made. I doubt very much whether the Government will entertain the latter idea, but, of course, they will supply both men and horses according to Lord Kitchener's needs. We shall, perhaps, know better next week, after Lord Roberts arrives, what the prospects of an early termination of the war really are. Meanwhile, I do not think that the croakers have spoilt the Christmas season for the vast majority of sensible people.

THE fact that a second lady in France has won her way to forensic honours will probably make English women more determined than ever to persevere until the same privileges are accorded to them as to their French sisters. A week or two back an English lady attempted to take the first steps towards becoming a barrister, but was informed that there was no precedent for such a course, and she had sadly to retire from the contest; at any rate for the present. But following hard upon the initiative of Madame Petit, Mademoiselle Chauvin has just taken her oath at the Palais de Justice. The ceremony was the occasion of quite a fashionable function, for the lady's friends and relatives and many who knew her only by reputation came down to the court to witness the admission. The lady is fortunate in having a brother also a member of the Paris Bar, which may, of course, have facilitated matters for her. She wore a gown made almost exactly like that donned by the men, but, woman-like, she succeeded, whilst apparently copying it exactly, in giving it quite an air of smartness. Under her cap it was possible to catch glimpses of a tortoiseshell comb which kept her hair neatly in place, but naturally she quite forgot to remove her cap, as did the other men, when interviewing the leader of the Bar in his private room. It must need a good deal of practice on the part of a woman to spontaneously uncover her head even in the presence of the highest legal luminary, and if, as one suspects, a bonnet-pin is used to keep the cap in place, the action becomes a little complicated and not easy to accomplish with grace. If Portias, however, become numerous, someone will speedily invent a "lawyeress' cap-clip" and solve the difficulty.

EVIDENTLY to lovers of Wagnerian music there is magic in the name of Bayreuth. Every year now there is a performance of the "Ring" operas in London, where the artistes are at least as good, if not better, than at Bayreuth, and where the scenery and lighting is superior. There is no denying that these performances are well attended, and of course if the prices were not of necessity as high as they are at Covent Garden, the number able to listen to the glorious music would be far larger. But still there is nothing like the enthusiasm over here which is manifested with regard to the Bayreuth performance. The first cycle of "Der Ring des Nibelungen" takes place there July 25–28, and yet the whole of the seats are booked already. There is small security to these enthusiasts that their position in the house will be such as they would wish, because even the seats are not allotted until March, so that all must be left to Fate, and Fate in the form of a bad corner with no comfort, £1 each night to pay for it, hundreds of miles to traverse, with hotel bills en route before getting to Bayreuth, hardly seems worth courting with such assiduity as English and Americans are now showing. But Fashion counts for much apparently even with ardent lovers of music.

TALKING of music, I must admit I have a good deal of sympathy with those dancers who are protesting against the dance music of the present day. Although many lovely waltzes have been composed by the best composers, they are now seldom heard. The strains to which the modern young man and young woman are invited to circle is usually made up of a few bars of some vapid air from the latest comic opera which has "caught on," frequently distorted from its original tune so that it may serve as a waltz, and "padded out" by a good deal of what can only by courtesy be called music at all. Perhaps it may be this want of incentive to dance in the shape of mirthful music which has resulted in the partial strike of young dancing men. They will oftentimes put in an appearance at a ball, more especially if the hostess is a favourite, or any of their "best girls" are to be there, but only a small proportion can be prevailed upon to dance more than a few times. Perhaps many unconsciously feel what a young fellow was heard to say the other day, "Bother this music. It makes me feel tired trying to find the tune in it." A lady giving a dance might try what result it would have on the masculine mind if she put "Waltzes by Gung'l, Waldteufel and Strauss" on her cards. I fancy she would find the percentage of acceptances from her male friends higher than usual.

THERE is a decided dislike, at least in England, to eating horseflesh in any form. The fact that the Royal Society for the Prevention of Cruelty to Animals has had to summons a horse dealer for allowing horses to go along the roads when they were quite unfit to work has led again to the defence, which has often been made before, that the horses in question were not going to be worked, but to be eaten. They were simply making their way to the sea-coast in the cheapest manner possible—on their own legs—and when they reached the water they would be taken across to the Continent, thence to return in a different form, in the guise of German sausage. Now it is quite certain that there is a large demand for this equine sausage, as it is cheap to buy and tasty to eat, and I know that it is a great favourite for supper in some kitchens, because the servants say that "it has so much more flavour than cold meat," meaning beef and mutton. Moreover, it is apparently wholesome enough, ill effects after a hearty meal being almost unknown, so that it must probably be prejudice rather than any firmer foundation which has caused the universal housewife to shudder at the notion of having such flesh in the house. But assuredly the testimony of a young officer who has returned from South Africa is worth something. He came back two stones heavier in weight than when he went out, and as he had been through the siege of Mafeking, his friends naturally inquired to what he attributed his fine form. His reply was "horseflesh sausages." He found them exceedingly nutritious. And it is said that General Baden-Powell had a similar experience, for when it was proposed to take a photograph of the officers in the besieged town as a memento of the privations they had endured, he stood at the back of the group, lest, in his own parlance, he "should give the show away." Perhaps if some enterprising tradesmen were tore christen the sausages, "the Baden-Powell" patriotism on the part of many Englishwomen and Englishmen would result in their becoming for a time not only the favourite food of "Jeames and the cook, but of master and his dinner guests."

### A Book and its Story.

### THE ROMANCE OF PLACES AND THINGS.

If it be true "that association is the poetry of romance," it is no less certain that association is the romance of real life.

Miss Coleridge[1] has written an historical novel with notable distinction, and she is also distinctive in her present book of "impressions" written in various places, and in varying moods.

Books should be like friends, real friends, never very far from us, ever ready to cheer, to console, and, if need be, to "counsel or condemn." Above all they should fit in to the mood or the occasion which makes their presence welcome, and then withdraw. Books, perhaps, have the advantage in being silent but endlessly suggestive companions, and one can put down a book at any moment. Not always can one thus treat a friend. "Non Sequiter" is a book of the suggestive interpretative class. As the authoress saw, as she felt, so she writes—with freshness, with vivacity, with a fine reserve.

The ground covered is neither new nor original. But it is trodden by one who moves with their inner eye awake. Kensington Museum, with reflections on its priceless collection of treasures, has many interesting pages devoted to it. Writing of "The Hall of Pillars," surnamed also "The Chamber of the Dead," of the indifference of all truly great souls to future fame, she says:—

"How many futile words have been said about the desire of men to perpetuate their names! If we may trust a popular writer, it does not exist in the finest natures of all, i.e. the most courageous. They tell us they do fine things because of it, but when the Nelson of the period cries out, 'Victory or Westminster Abbey!' Westminster Abbey goes for very little, and he fights because he enjoys fighting, and would give battle in the same spirited manner if he knew that he was sure to go to the bottom, and the action won and he never be heard of. Heroes have no wish to be rembered. Indeed, to a practical man the present is such a festival of speculation that he has no room for the future. He sees around him work which in his opinion he is the only person fit to do—work which he is aware from the nature of things he can never hope to accomplish."

Unlike the future, the past, with all the lessons to be learnt from its failures, its successes, is a *field* that no sensible man can afford to ignore. "He may hate it with the energy that a good son feels for a bad father, but there are certain lessons that he *must* learn from it before he can understand his own life and the lives of those around him. From the category of "the finest natures of all" the authoress excludes the poet. He having a weakness for epitaphs, and being of those who imagine that, as they are not understood in their lifetime, they may hope to be after their death. "They therefore wish to be remembered, and are in this unlike heroes, but not unlike prophets and martyrs, who live nowhere if not in their own fame after death. Poets, prophets, and martyrs have a certain ground of common imagination, and interchange characters oftener than other people; but the two last are not so outspoken as the first in personal matters.

This is ingenious humour, and rather hard on the poet, who, after all, is a being who of necessity *sees*, and therefore feels, acutely, otherwise were he not able to speak to the hearts of others. Prophets and martyrs are of sterner stuff. Prophets are also, primarily, seers, and they preach endurance, not of necessity practised, personally. To the martyr is left the highest perfection of heroism, not only to endure and to suffer, but to die for the faith that is in him.

Perhaps there is no more common error than that associated with the use of the word "Mizpah." How few people realise the origin of the word when exchanged as a token of fidelity. How few realise that its significance has a purely *business* origin, in reference to transactions between Jacob and Laban, who, with usual Eastern astuteness, begged that "the Lord would watch between them" in order neither should take advantage of the other during his absence. Miss Coleridge is remarking on the silence of a cemetery; its reserve, in some instances also its abandon of unreserve, as shown in its voluble epitaphs. In this one word "Mizpah" brevity holds her own, and if only the bereaved ones realised it, would it be possible for such an inscription to be used to express the immortality of conjugal love? "We may be very sure that Jacob never put it on the first monument—the pillar set up in memory of Rachel."

She has a word on travelling companions, books and people. The former are "a terrible responsibility." "We cannot be sure of taking anyone with us, even our dearest friend; he may be half a realm away the whole six weeks for all we know. A peevish anxiety to reach the post office betrays him. . . . Selection is but a vanity, chance is as good as choice, and better." The impressions of well-known places like Cologne, Homburg, Frankfort, are all brightly discursive reading seen through the practical imaginative mentality of the authoress. They conclude with, "It is with places as with people. Some are charming when they are young, and never at any other time; while others improve in middle age; and others again not until the coming on of years has bent and wrinkled them. Some few—and these are rarest—are lovely all their lives. It is difficult to conceive how Venice can ever have been otherwise. Manchester, on the other hand, will not be lovely if she lives to eight or nine hundred."

Shakespeare, for instance, will have his revenge if you leave him behind. How true it is "that there is no mood in which he cannot be read. It may be only a sonnet that is wanted; but nobody else's sonnet will do instead." Then also, "If it be a safe and simple rule not to quit England without Shakespeare in the bottom of the box, it is an equally important point of the law of enjoying oneself never to travel without half a dozen novels in the top of it." These novels must be good, and they must be long, as they have to beguile the hours through miles of uninteresting country, and their goodness must be of the first order, worthy of being read more than once. As a contrast, Wilkie Collins and George Meredith are suggested—the one because it takes so little time to understand him, the other, for obviously diverse reasons. After all, the conclusion arrived at is that books are more satisfactory as travelling companions than individuals. They, at any rate, don't bore you or argue with you. They do not fail you at the moment when you require them, or thrust unwelcome attentions on you when you desire repose."

There is a sad little picture of Mrs. Fanny Kemble among the recollections of people, as she was found by Miss Coleridge "sitting alone at her window, dressed in black velvet, busy with the most hideous bit of worsted work in the world. I do not remember any of the colours in it except that the worst and the most prevalent was violet. She looked as if she were going to cry—very sad—very old." "Non Sequiter" is full of unstudied charm, and it will find many appreciative readers among those who value fugitive thoughts put into good English in a style at once refined and cultivated.

[1] "Non Sequiter." By M. E. Coleridge. (Nisbet, 6s.)

### Everybody's Opinion.

[Correspondence on all subjects is invited, but we cannot in any way be responsible for the opinions expressed by our correspondents. No communication can be entertained if the name and address of the correspondent is not given, as a guarantee of good faith but not necessarily for publication, or unless one side of the paper only is written on.]

### NORTH DEVON INFIRMARY, BARNSTAPLE.

"ONLOOKER" writes: May I give a word of warning to any trained nurse applying for a vacancy at the North Devon Infirmary? The vacancy advertised recently was only filled three weeks ago; but the nurse will not stay, the reason given being that the duties are totally different from the agreement, and more than one nurse could possibly do. This state of things has been going on for some time. The nurses' rooms are stated to be inadequate for health, and not to possess the smallest degree of comfort.

### RULES IN PRIVATE NURSING.

"A PRIVATE NURSE" writes: It is a very difficult matter to decide whether a patient should be woke up for his or her medicine at the proper time, as every case varies, and in this one has to use their own discretion as to whether sleep or the medicine would prove more beneficial. As regards sleeping in a convalescent man's room, again circumstances, I should say, would alter cases; and I do not think we should be credited with being immodest if we were compelled to do so; but in most private houses I have found that one can generally come to some satisfactory arrangement.

"SISTER G." writes: It is almost impossible to lay down any rule in private nursing. "A Watcher" should ask her doctor what he wishes done about giving the medicine to her patient: if he is to be woke (as many have to be) at the regular hours, or allowed to sleep until he wakes himself, and then have the medicine given. It is always best to consult the doctor and keep everything written for him to read. It is not usual for a nurse to sleep in a convalescent man's room, and I strongly recommend "A Watcher" to refuse to do so if ever it is proposed to her. She could sleep in a room close at hand, and go in once or twice during the night, and if she is a heavy sleeper get some cord, fasten it to her patient's bed-post, slip it through his key-hole, then through her's, and hang a small hand-bell on the bed in her room, so that if her patient required anything, he could ring her up. The sooner that "Matron" is asked to resign for the sake of the nurses in her institution the better; no wonder they get a bad name when their matrons are so lax.

"DUTY" writes: It is always best to ask the doctor in attendance if he wishes the patient to be awakened to take medicine or food, as it is often ordered to be taken at regular times. Certainly the time each thing is taken should be written down, and the quantity of food or stimulants, also how long the patient sleeps, and if quiet or restless when asleep. It is for each nurse to decide for herself if it is right to sleep in a patient's room, whether that of a man or a woman. If a nurse is old enough to nurse a man in a private house she ought to be old enough to sleep in the same room, if the necessity arises. Of course, there could be a screen between the patient's bed and the nurse's, unless, as is often the case, the latter has a small one at the foot of the patient's. If the arrangement is unpleasant for the nurse it is also unpleasant for the patient.

### A CRY FROM WHITBY.

"THE Honorary Secretary of the Whitby Cottage Hospital and District Nursing Association" writes: A copy of your paper of the 15th inst. has been sent me containing an article entitled "A Cry from Whitby." On behalf of the Whitby Cottage Hospital Committee I shall be glad to know your authority for the statements contained in the aforesaid article. The Bank Buildings, the purchase of which was decided upon unanimously at a public meeting, were selected as the site for the new Cottage Hospital on account of their central, quiet, and sunny situation, and have been thoroughly examined and approved of by several of the local medical men; also by two London medical men, both Fellows of the Royal College of Surgeons. All the medical men of the town and district are members of the General Committee of the hospital, and their opinion is probably of more value than that of your informant. It has been decided to pull down the old part of the Bank Buildings in order to build commodious bath-rooms, kitchens, &c. The wards, nurses' sitting-room, &c., command a lovely view of the harbour, and get all the sunshine obtainable; there is a large open yard in front of the house, and the soil is gravel. The present tenants, who are leaving the house with much regret, have never had water in the cellars or any illness in the house, excepting measles, during their seventeen years' tenancy. I shall be glad to give you any further information, and I think you will admit that the statements in your article upon this question are not justified by the facts.

[We are not in the habit of disclosing the sources of our information, but we may say that the "Cry from Whitby" came from someone who claims to know the circumstances of the case, and to express the feelings of many people in Whitby. Of course, we did not endorse the statements made to us, but merely insisted that the situation of the permanent cottage hospital should be unimpeachable in respect to healthiness. Much better to thresh out a matter of this sort before building a hospital than afterward.—ED. HOSPITAL.]

### NURSES' READING SOCIETY.

"MISS MOBERLY" writes: I regret to say that we have had a considerable falling off of members lately—our numbers are now only 40. Some of the members who have left us have written to explain that press of work, or some other excellent reason, has prevented their remaining in the Society. But many members have made no communication to me whatever, though their subscriptions were due on July 1st. Having given them some months' grace, I have now struck them off the list, with those whose subscriptions were due in October and who have not paid them. Should any of these nurses desire to rejoin the Society I shall be happy to re-enter their names. I have received from many old members most warm and cordial letters, expressing their gratitude for, and appreciation of, the use the Society has been to them. I should like to take this opportunity of thanking them for their kindly support and encouragement and of wishing them all a happy new year.

### "VISITORS FOR THE UNVISITED."

"A NURSE" writes: The question of visitors for the unvisited patients of our hospitals and infirmaries is attracting some attention in the daily press. "Visiting day," in our hospitals comes round to the sister and nurse as a not entirely unmixed blessing. Although, to wish to keep away anyone near or dear to a sick person may seem to many cruel—and, indeed, it is so in some ways—perhaps only a doctor or a nurse can know the damage which is often done to a patient by well-meaning but injudicious visitors. What nurse does not remember, in her probationer days, the agony of mind she suffered when set to watch the "typhoids," during visitors' hours, that they might not receive surreptitious gifts of food, &c.? In fact, any band of untrained visitors—whether relations or not, whether educated or otherwise—no matter how excellent their intentions, could not help something causing the evil that is wrought by want of thought. But when it comes to the visiting of children, the case becomes somewhat different. Many an educated girl who finds time hanging heavily on her hands might devote an hour or so each week to visiting and reading stories to the convalescent children in our hospitals, and with good results. Convalescence is very tedious to children, and it is seldom that hospital nurses have time to amuse or play with the little ones, much as they would at times like to do so. A visitor who is a near relation often upsets a child, and "Baby Ward" for some time after "visiting" hours is generally a scene of many tears and frantic cries for "Mummy" and "home." There are not many mothers who would forego the pleasure of seeing and speaking to their children—though the latter might gain very little benefit thereby—even if they realised the harm done. The most beautiful example of a mother's self-sacrificing devotion in this respect that I have known was in the hospital where I was trained. One day, during visiting hours, I noticed a woman standing in a dark passage by the open door of one of the children's wards. I went up to her and asked her what it was she wanted. "Oh, I've come to see my little boy"—mentioning one of our favourite children. "Then why don't you come in?" I asked in astonishment. "No, Miss," said the mother. "I did that once, and it upset him so that I would not do it again. I know it makes his eye worse for him to cry so; and I do want him to get well quickly. Home is a dull old place without him! So every visiting time I come and stand in this passage, where I can see him but where he can't see me." She was a poor woman, and he was her only child—"a beautiful boy of three years old."

### NURSING ARRANGEMENTS IN SOUTH AFRICA.

"A SISTER OF THE A. N. S. R." writes: Your correspondent in the "HOSPITAL" NURSING MIRROR of October 6th seems to wish for one of two things—either that the standard for entering the Army Nursing Service be raised from that of the three years' certificate, or else that the sisters should take more the position of a staff nurse with a senior sister to overlook them in a group of wards or tents. I feel very strongly that the latter suggestion would, if carried into effect, prevent many very excellent sisters of experience in ward management from volunteering in a time like the present. In my own hospital of 200 beds out here in Natal we began work with six sisters of the A. N. S. R., none of them with less than five years' experience. Since then we have had several changes of station, and our staff now numbers nine sisters. The six vary in nursing experience from one of twelve years' work in our best English and Scottish hospitals to one who has just gained her three years' certificate. Our superintendent, in spite of having "to housekeep" (not a very serious business out here) and "to struggle with native servants" (we have no maids with us)—I quote from your issue of October 6th—can find time to visit each ward at least once a day to discuss any little difficulty the sister may be experiencing in getting what she wants for the comfort of her patients, besides frequently coming in at odd times to help the men with their knitting or worsted work and keep up their supply of materials. Of course, in a much larger hospital it would be more difficult for her to go all round every day, but would it not be better for her to take a junior sister to help her with the housekeeping and some of her routine work, rather than to allow the actual superintendence of the nursing to pass out of her own hands? For however senior a ward sister may be, she would be willing to discuss her difficulties with the lady superintendent; the assistant could be appointed for a fortnight or a month at a time, and then be replaced by another. Of course there are generally large and small wards, also acute and convalescent wards, and a superintendent would naturally not place a sister who has had no experience in ward management in a large and important ward until she has tested her capabilities in a less responsible post and helped her with advice as to the way things are managed in a military hospital. Another point to be considered is that a ward sister in a military hospital does not hold such a responsible post as a sister in a large civil hospital, I was trained at one of our largest London hospitals, and was a sister there for five years, during which time I had charge of both medical and surgical wards; besides being responsible for the training of all the nurses who passed through my ward, for the care and treatment of the patients, for seeing that all the directions of the medical officers were carefully carried out, and for the ordering of the diets and stimulants and seeing them served. I was also held responsible for the care of the ward itself, that the floors, &c., were properly cleaned, the chimneys swept when necessary, that all breakages were repaired or replaced, and I had likewise charge of the linen. I had to report the admission of each patient and obtain the necessary cards, &c., from the office, to keep a careful register of all cases, with full particulars of the dates of admission and discharge, the diagnosis, operation, &c.; to arrange about the transfer of suitable cases to convalescent homes, and to see that they were properly fitted up with clothes; to report any death that might take place to the office and do the necessary correspondence. How different is the work of a sister in a military hospital! To take my points in order: the sister is responsible for the training of the orderlies in her ward, for the treatment of the patients, &c., and the giving of all medicines and stimulants, but the ordering and serving of the diets is entirely in the hands of the senior orderly and the ward master, and they are also responsible for the cleanliness of the ward and the care of the ward furniture and fittings. The linen is not kept in the ward (with the exception of a few extra sheets, &c.), but is issued from the store as required. When patents are admitted (we have sometimes had 100 in at a time) the sergeant goes round and fills up their papers with name, rank, regiment, &c.—the sisters have no book-keeping to do or registers to keep—they take away their kits to the store and their valuables to the office. When men are leaving it is all arranged for them through the office; if they need clothes the ward master has to obtain them from the quartermaster; if they need their dinner early before starting the ward master has to arrange for it; if a death occurs you send for the ward master and need take no further steps. As a matter of fact, I always try to obtain the home address of any poor fellow who is likely to die and write to his friends, and many a grateful letter have I had in consequence, but this is not considered a necessary part of a sister's duty. I always felt in my London hospital that my ward was my home and that I was not only personally responsible for the comfort, health, and training of every nurse and patient, but also for the ward being kept as clean and nice and made as homely as I would wish a home of my own to be. You cannot have the same feeling in a military hospital, where so much is taken out of your hands; and I think that, with a good superintendent to overlook and advise, a lady who has had three years' training in a good hospital, should be well able to do the nursing and all the superintending that is required of an Army sister. I do not for a moment wish to imply that I think sisters with longer experience are wasted in the army nursing service, especially in time of war. I only desire to appeal against its being thought necessary to appoint superintending sisters in addition to the one lady superintendent, and thus check sisters of long training, who might not care again to take the lowest place, from coming forward.

### Artistic Novelties.

JOHN HARRIS AND SONS are showing at their art embroidery show rooms, 250 Old Bond Street, the most delightful assortment of useful and beautiful articles suitable for Yule or birthday presents. Their flax embroidery linen has long been famous throughout the artistic world for the delicacy and purity of its colouring. Here we find it worked up into the most ravishing novelties of various kinds, some of which are worthy of special notice. To nurses I desire to recommend a charming tray sufficient for a cup and saucer or soup bowl, in delicate white china, covered with a cloth of pale blue and artistically worked in a floral design, costing half-a-guinea; also a scribbling diary interleaved with blotting-paper, covered with different coloured linens with suitable design worked on the back for the sum of 11s. 6d. I likewise admired a beautifully worked handkerchief sachet, which on inquiry I found was for sale at 4s. 6d. A pocket diary for 1901 is another attractive novelty, a few copies of which still remain at 1s. 6d. Table covers and couvre-pieds there are in abundance, and in the most lovely shades and designs. The same article can be purchased ready traced for those who are industriously inclined, and at a reasonable price. Plain linen by the yard is procurable for those who are artistic enough to make their own designs, and samples will be sent on application. If any of my readers feel disposed to call and see the courteous manageress next time they are in Bond Street I feel assured they will consider both time and money well invested in some of the beautiful articles which are so lavishly displayed.

### Our Christmas Distribution.

THE generous contributors to our Christmas distribution of clothing will read with interest the following letters which we have received in acknowledgment of the parcels sent out:—

THE MATRON of King's College Hospital writes: "Please accept our grateful thanks for the warm clothing so kindly sent for our poor sick when leaving the hospital, and which will be greatly appreciated by them."

THE MATRON of the Victoria Hospital, Chelsea, writes: "I beg to thank you for the toys you have kindly sent us."

THE MATRON of the Metropolitan Hospital, Kingsland Road, writes: "Thank you very much for your very useful gift which came to-day; we are most grateful for the warm clothing you have sent us, everything is so good and serviceable, and we appreciate your kind remembrance of us very much. It is such a help to have a few warm garments to give to the poor convalescent patients when they leave us to go to their miserable homes."

THE MATRON of the Maternity Charity and District Nurses Home, Howard's Road, Plaistow, E., writes: "Very many thanks indeed for the parcel of nice clothes you have so kindly sent to me. I am most grateful for them; they are very acceptable just now."

THE SUPERINTENDENT of the East End Mothers' Home, 396 Commercial Road, Stepney, writes: "I beg to thank you and your readers for the very nice parcel of clothing just received. My patients, I know, will greatly appreciate your kind and pretty gifts. There are ten mothers in the Home, six of whom will be here for Christmas; and as there is a long list of women waiting to come in, we hope to have the Home quite full."

THE SECRETARY of Charing Cross Hospital writes: "I am directed by the Board of Governors to convey to you their sincere thanks for your kind gift of clothing for the patients."

THE MATRON of St. Vincent's Hospital, Gort, Co. Galway, writes: "I most gratefully acknowledge, with my best thanks, a parcel of warm clothing received from you."

THE MATRON of the Royal Hospital for Children and Women, Waterloo Bridge Road, writes: "I have to acknowledge with many thanks the warm clothing sent for the patients. They will be most useful."

THE SECRETARY of the East London Hospital for Children and Dispensary for Women, Shadwell, writes: "I beg to acknowledge the receipt of your letter of the 17th inst., and on behalf of the Board of Management to thank you very sincerely for your welcome present of warm clothing, which will be much appreciated by the children on leaving the hospital."

### Appointments.

HERTFORD BRITISH HOSPITAL, PARIS.—Miss Sara S. Neech has been appointed Matron. She was trained at Guy's Hospital and the Royal Infirmary, Glasgow. At the latter institution she was charge nurse for some time. During the past three years she has held the position of matron at the Memorial Hospital, Jarrow-on-Tyne.

FOUNTAIN FEVER HOSPITAL, TOOTING GRAVENEY.—Miss Jessie Charlotte Savile has been appointed superintendent of night nurses. She was trained at York County Hospital and has since been charge nurse at the Fountain Hospital.

### Minor Appointments.

ROYAL HALIFAX INFIRMARY.—Miss E. M. Smith has been appointed sister. She was trained at Burton-on-Trent General Infirmary and has since been charge nurse in the same Institution, sister at Eston Hospital for Accidents, and sister at Grimsby and District Hospital.

NEW UNION INFIRMARY, WARRINGTON.—Miss Fanny Watson has been appointed charge nurse and assistant superintendent. She was trained at Chorlton Union and has since been charge nurse and midwife at the Stoke-on-Trent Union Hospital. She holds the L.O.S. certificate.

### Not Divided.

ON a hospital bed she was lying
Her face was drawn with pain;
"Oh nurse! let me see my baby
Once again.

"The world has been cruel to me, nurse,
Yet I should have liked to stay,
For I know 'twill be hard for my baby
All the way.

"But if I could have her here, nurse,
And clasp her once more to my breast,
Perhaps I might die more peaceful
And could rest.

"But oh! nurse! bring her quickly,
I feel I am going now!"
And the cold drops gathered thickly
On her brow.

The nurse's eyes were tender
As she smoothed the poor damp hair;
"You won't be lonely in heaven, dear,
Baby is there."

### Working Boys.

MR. STUART M. SAMUEL M.P., presided over a pianoforte and vocal recital given last week by Mr. George Denham at the Bishopsgate Institute on behalf of the Homes for Working Boys in London. In his opening remarks Mr. Samuel said he was anxious to show sympathy with the good work which is being carried on by this society, two of the homes being in the borough of Whitechapel, which he represents in Parliament. Mr. Samuel considered that the homes intended for honest and industrious boys were really a mother to the boys, and under the excellent superintendents who serve the home a boy receives those good influences which a boy's early home should give. No better means, he urged, could be used for providing for a boy's future than by making him God-fearing. No matter to what religion he might belong that boy would be a good citizen. Mr. Samuel pleaded for contributions towards fitting up the gymnasium in the home in Spital Square, and added that he was very willing to contribute his share in support of the excellent work. Mr. George Denham was well received in his arduous task of some nine songs and two pianoforte solos. He was ably assisted by Madame Amy Young in a clear and telling voice and by Miss Batchelor, who was most favourably received. Master G. T. Denham showed considerable command over the keyboard for one so young, and his rendering of Weber's "Moto Continuo" and Mendelssohn's "Rondo Capriccioso" were most praiseworthy.

CHRISTMAS BOOKS.—"The Bag of Diamonds and Three Bits of Paste," reviewed in our last issue, is published by Messrs. Chatto and Windus, Piccadilly.

### For Reading to the Sick.

NEW YEAR'S EVE.

"WHERE meets the dying and the dawning year?"
"Faithful unto death."
Art thou too weak to pray?
Then, spirit, rest;
Lie where St. John lay, on thy Master's breast,
He knows thy weakness, understands each sigh,
The yearnings of thy heart, its voiceless cry.
A child who knows not why nor whence its pains,
But meekly lies and frets not, nor complains,
Is as a dewy flower that breathes at even
A perfume sweet, into the heart of Heaven.
Lie, child, like thou, and ask not whence nor why,
Lie still and hear thy Saviour's lullaby.—*C. M. Noel.*

Where He is is great peace. Learn to commune with Him in stillness, and He whom thou hast sought in stillness will be with thee when thou goest abroad.—*Pusey.*

O Jesus, Thou Healer of the sick, we impotent folk draw near Thee to-day bringing our sins and sorrows, our weakness and our faults, our broken resolutions, our many failures. Speak the word only, and Thy servants shall be made whole.—*H. W. B.*

Remember, my soul, that thou art but a passenger in this world: do not place thy affections on what thou seest; look and pass on and seek for thyself a good home where thou mayest dwell for ever.

O ye past and bygone years, what witnesses ye are of my soul's waste and forgetfulness of God! O Jesu, obtain for me through Thy mediation the gift of final perseverance, that I may endure to the end, in grace and love, until I am fit to enter Thy heaven above.—From *Devotional Helps.*

Here we all suffer, walking lonely
The path that Jesus once Himself hath gone;
Watch thou this hour in truthful patience duly,
This one dark hour before the eternal dawn.

And He will come in His own time from Heaven
To set His earnest-hearted children free;
Watch only through this dark and painful even,
And the bright morning yet will break for thee.
*Anon.*

O Lord our God, who art in every place, from whom no place nor distance can ever separate us: we know that those who are absent from each other are still present with Thee, and we therefore pray Thee to have in Thy Holy keeping those dear ones from whom we are now separated; that whether or not, according as seemeth best to Thy Divine Majesty, we meet again here on earth. We may surely meet again at the resurrection of the just, and go in together to that house of many mansions which Thou hast prepared for those that unfeignedly love through Jesus Christ our Lord. Amen.—*Anon.*

Suffering is the work now sent,
Nothing can I do but lie
Suffering as the hours go by;
All my powers to this are bent.
Suffering is my gain; I bow
To my Heavenly Father's will,
And receive it hushed and still;
Suffering is my worship now.
From *Lyra Germanica.*

### "The Hospital" Convalescent Fund.

THE hon. sec. has much pleasure in acknowledging the receipt of 7s. 6d. from Miss K. Pride with grateful thanks.

### Notes and Queries.

**The Editor is always willing to answer in this column, without any fee, all reasonable questions, as soon as possible.**
**But the following rules must be carefully observed:—**
1. **Every communication must be accompanied by the name and address of the writer.**
2. **The question must always bear upon nursing, directly or indirectly.**

**If an answer is required by letter a fee of half-a-crown must be enclosed with the note containing the inquiry.**

*Maternity Fees.*

(141) I was engaged to nurse a maternity case in August, and consequently refused others for that month. The confinement being premature, I was unable to attend. Can I claim any fee?—*Cecilia B.*

It is customary to allow half fees in such an instance; but your claim should have been made when the child was born. You must safeguard yourself in future by giving each prospective patient a card with your scale of fees.

*Probationer.*

(142) Will you kindly tell me of a children's hospital which accepts probationers of eighteen? I wish to receive payment.—*E. E. B.*

You are too young for any as yet. See the "Nursing Profession: How and Where to Train" for full information on the subject.

*Insurance.*

(143) Would you kindly tell me of any insurance against accidents and sickness? I would be very grateful.—*Private Nurse.*

You should communicate with the Secretary of the Royal National Pension Fund for Nurses, 28 Finsbury Pavement, E.C., with a view of becoming a member.

*Maternity Fees.*

(144) A lady engaged me for the end of November on the understanding that the confinement might take place in London or elsewhere. A fortnight later she cancelled the engagement. As I refused another engagement in that time can I claim, by legal measures, any compensation for my loss of fees? The engagement was verbal.—*Monthly Nurse.*

It is not customary to claim compensation if the engagement is cancelled within three months from its commencement; besides a verbal agreement is worth nothing.

*Lying-in Hospitals.*

(145) Kindly tell me which is the best lying-in hospital for training in maternity work for a nurse wishing to obtain cases in London and the provinces.—*I. C.*

Queen Charlotte's Hospital, Marylebone Road, N.W., would probably suit you.

*Twenty-one.*

(146) Are probationers taken in any hospital at twenty-one? If so, will you kindly give me the address?—*K. P.*

See lists of children's and of special hospitals in the "Nursing Profession: How and Where to Train."

*Maternity Training.*

(147) J. C. is desirous of obtaining maternity training in London if possible. Which hospital is the best and cheapest?

See answer 145 above. The City of London Lying-in Hospital, City Road, E.C. (fee for pupil nurses £7 7s.); the British Lying-in Hospital, Endell Street, E.C. (fee £7 3s.); the East End Mother's Home, Commercial Street, E., are all excellent and very cheap.

*Massage.*

(148) Would you kindly tell me where a young man could learn massage and electricity; and what the fee would be?—*Henry G.*

At the National Hospital for the Paralysed and Epileptic, Queen's Square, Bloomsbury, W.C. Write to the Secretary for scale of fees.

*Probationer.*

(149) Madame M. would feel obliged by the Editor sending her particulars of country convalescent homes for children; and of the age at which probationers are received.

There are no lists published of convalescent homes which train probationers. There is a convalescent home for children at Loughton, Essex, where probationers are taken, and also the Children's Cottage Hospital, Coldash, Newbury, Berks.

*Blushing.*

(150) Would you kindly tell me if there is a cure for blushing; if there are any books published upon the subject; and if, there is no way of hardening the skin?—*I. G. M. H.*

Blushing is of nervous origin, and if the subject is otherwise healthy the cure is best accomplished by self-forgetfulness and self-control.

*Appointment.*

(151) Would you be so kind as to inform a nurse-attendant (untrained) how she could get a post in Algiers, or in the South of France?—*Miss R.*

By advertisement. Read those under the heading of "Appointments Vacant" in the MIRROR.

*Address.*

(152) "Nurse" would be glad to know the address of the After-care Association for persons discharged from asylums.

Church House, Dean's Yard, Westminster, S.W.

**Standard Books of Reference.**

"The Nursing Profession: How and Where to Train." 2s. net; post free, 2s. 4d.
"The Nurses' Dictionary of Medical Terms." 2s.
"Burdett's Series of Nursing Text-Books." 1s. each.
"A Handbook for Nurses." (Illustrated.) 5s.
"Nursing: Its Theory and Practice." New Edition. 3s. 6d.
"Helps in Sickness and to Health." Fifteenth Thousand.
"The Physiological Feeding of Infants." 1s.
"The Physiological Nursery Chart." 1s.; post free 1s. 3d.
"Hospital Expenditure: The Commissariat." 2s. 6d.

All these are published by THE SCIENTIFIC PRESS, Ltd., and may be obtained through any bookseller or direct from the publishers, 28 and 29 Southampton Street, London, W.C.

### Travel Notes.

By Our Travelling Correspondent.

### LXIV.—SIX MONTHS IN ROME.

### The Royal Requiem Mass in the Pantheon.

To attend this a *permesso* must be obtained from your banker. King Victor Emmanuel was buried in the Pantheon twenty-two years ago, on the 12th of January. The building is the most ancient religious structure in Rome in a state of preservation. Though begun twenty-seven years before our Lord's birth, the greater part of the building dates from Hadrian's time. It was consecrated as a Christian Church in 608 by Boniface IV. The only light comes from a circular opening at the top of the dome, which gives a very singular effect.

"I think . . . it is to the aperture in the dome—that great eye gazing heavenward as it were—that the Pantheon owes the peculiarity of its effect. It is so heathenish, and so unlike all the smugness of our modern civilisation! Look at the pavement directly beneath the open space! So much rain has fallen here in the last 2,000 years that it is green with small fine moss, such as grows over tombstones in damp English churchyards."

"'I like better,' replied Hilda, 'to look at the bright blue sky roofing the edifice where the builders left it open . . . . would it be any wonder if we were to see angels hovering there, partly in and partly out, with genial heavenly faces, not intercepting the light, but transmuting it into beautiful colours? Look at that broad golden beam—a sloping cataract of sunlight, which comes down from the aperture and rests upon the shrine. . . .'" Transformation.

It was to this magnificent temple of the past and present that we wended our way, provided with the necessary tickets. The Piazza was kept clear by carabinieri, and the doorway was approached through an avenue of infantry. Inside, we found ourselves in a kind of roped-in pen, provided with chairs. In the centre, under the great eye, rested the enormous catafalque three storeys high, with a canopy over it, and candles and funeral lamps all round. I should mention that the aperture for lighting was covered with black cloth, so all the illumination was artificial, and the effect most sombre and grand.

At the foot of the catafalque enormous wreaths rested, and at each corner mighty candelabra. Presently in marched thirty most lovely cuirassiers, such magnificent creatures in long boots, tight white breeches, and dark blue tunics; over these last, resplendent cuirasses under the edges of which scarlet pads appeared, steel and gold helmets with black horse tails and red and white bottle brushes—in fact "everything handsome about them." The officer was gorgeous to behold, though a little stout for the style of dress. They stood in a line each side of the gangway, and eight kept guard at the corners of the catafalque. The officer walked up and down with his sword drawn, and when a superior officer came in, he saluted by swishing it in the air. It was interesting to observe that these beautiful persons kept their pocket-handkerchiefs in the tops of their boots, whence they drew them forth and blew sonorous noses, quite like common folk!

The mass began at ten, and the music was very fine, and the effect of the muffled drums especially grand. Towards the end a priest in a black and gold cope went round and censed the catafalque, whilst the choir sang exquisitely. It was a splendid sight, and one on no account to be missed. Now, alas! there is a second royal death to commemorate.

### Carnival in the Corso.

It is a melancholy fact that the carnival in Rome is not nearly so gay as in Nice, but the surroundings, notably in the Corso, make up for the very inadequate procession and indifferent masks and dominos.

We first took tickets to see the races in the Piazza del Popolo, which for the occasion is turned into a kind of arena, and thickly strewn with sand. At 4 o'clock a gun was fired, and five riders called "Butteri" or campagna herdsmen came in. They trotted round to show themselves to admiring friends, and we picked out a little bay horse as a likely winner. There was difficulty in getting them into line. As soon as three or four were placed, the rest started aside to the aggravation of the starter, whose violent waving of his flag was the chief cause of their restiveness. Once off they tore round three times, the bay winning easily. The successful competitor raced again with the second winner, and was again successful, when he was presented with a large blue banner, which he carried round as a sign of triumph. His horse became violently excited—the flopping of the folds about his ears, the shrieks of the crowd, and the blaring of the trumpets were too much for his equanimity; at last his rider grabbed the flag in the centre and galloped round full pelt, and then pitched the offending blue drapery down as if he was very glad to be rid of it.

Then came a race between five of the "Rioni," or Wards of Rome. The riders were dressed in brilliant colours, the costumes being of the fifteenth century, with long hanging sleeves, &c. A gorgeous cavalier in blue, who finally rode his horse bare-backed, was the winner; and after this we retired to refresh nature and prepare for the evening.

We secured a good window in the Corso for the procession and confetti-throwing, and saw excellently; but the procession was poor. One of the cars had a colossal statue, representing Rome as the head of the world; but the top was jolted off, and the head of the world had to proceed minus her own.

### The Veglione.

The Veglione, or masked balls, at the theatres, are amusing and worth seeing as something quite outside our English experience, but you must not venture to go on to the stage without a gentleman—it would not be correct; but you can look on from the safe seclusion of a box if you are ladies alone.

The fun was perhaps a little tumultuous, and when the men could not get partners they danced with each other There were all kinds of dresses, but Pulcinello was very much in evidence as usual—a pair of white trousers and a nightshirt worn like a smock frock constitute the main features of the costume, also a white conical hat with which Pulcinello belabours the crowd. One of these gentry had, I think, found dancing thirsty work, and became exceedingly obstreperous, carrying off his partner bodily in his arms, whilst she kicked and slapped his face vigorously. A little of the Veglione goes a long way, but it is a curious sight, and may be viewed even at close quarters with perfect propriety if you are suitably attended.

### TRAVEL NOTES AND QUERIES.

Monte Carlo (Target).—You would find moderate hotels at La Condamine, which joins Monte Carlo, and is, in fact, part of it—Beau Séjour, Etrangers, and of a humbler kind the Monegasque. Considering your purpose, why do you not stay at Villefranche or Beaulieu, about three miles distant; constant trains. There is no reason why you should not stay at Monte Carlo, but I think you would be much more comfortable at Villefranche, which is an exquisite spot and cheaper than Beaulieu.

South by a Fresh Route (Alma).—Yes, there is a very pleasant way of reaching the Riviera by Puget-Théniers, *viâ* Lyons and Grenoble. The scenery is magnificent, and certainly superior to the better route *viâ* Marseilles. It takes considerably longer: you must stop at Grenoble and Digne. I find very few travellers know this route, because usually people are so anxious to reach their destination, and think nothing of the beauties they are passing unseen. The rail from Puget-Théniers to Nice is truly magnificent.

